# MDR *Escherichia coli* carrying CTX-M-24 (IncF[F-:A1:B32]) and KPC-2 (IncX3/IncU) plasmids isolated from community-acquired urinary trainfection in Brazil

**DOI:** 10.1016/j.bjid.2022.102706

**Published:** 2022-10-11

**Authors:** Juliana Buck Dias, João Gabriel Material Soncini, Louise Cerdeira, Nilton Lincopan, Eliana Carolina Vespero

**Affiliations:** a Universidade Estadual de Londrina, Centro de Ciências da Saúde, Departamento de Patologia, Análises Clínicas e Toxicológicas, Laboratório de Microbiologia Clínica, Londrina, PR, Brazil; b Liverpool School of Tropical Medicine, Department of Vector Biology, Liverpool, United Kingdom; c Monash University, Central Clinical School, Department of Infectious Diseases, Melbourne, Australia; d Universidade de São Paulo, Instituto de Ciências Biomédicas, Departamento de Microbiologia, São Paulo, SP, Brazil

**Keywords:** *Escherichia coli*, Urinary tract infection, CTX-M-24, KPC-2

## Abstract

Acquired antibiotic resistance in bacteria has become an important worldwide challenge. Currently, several bacteria, including *Escherichia coli*, have multidrug resistance profiles. Genes such as bla CTX-M-24 and bla KPC-2 (carbapenemase) are widespread. This research letter reports about a genomic surveillance study where multidrug-resistant E. coli containing CTX-M-24(IncF [F-:A1:B32]) and KPC-2(IncX3/IncU) plasmids were obtained from community- acquired urinary tract infection in Brazil.

Acquired antibiotic resistance in bacteria has become an important worldwide challenge.^1^ Currently, several bacteria, including *Escherichia coli*, have multidrug resistance profiles.^2^ Genes such as *bla*_CTX-M-24_ and *bla*_TEM-1B_ that encodes extended-spectrum beta-lactamase (ESBL) are widespread in *E. coli* strains.^2^^,^^3^ These genes can suppress the action of cephalosporin antibiotics.^4^ Besides, the presence of *bla*_KPC-2_ (carbapenemase) gene in the bacterial genome may confer a carbapenem resistance profile.^5^^,^^6^ During a genomic surveillance study, a multidrug-resistant *Escherichia coli* containing CTX-M-24 (IncF [F-:A1:B32]) and KPC-2 (IncX3/IncU) plasmids was obtained from community-acquired urinary tract infection in Brazil. This microorganism was isolated in 2016 from a urine sample from a community 79-year old female patient, without previous hospitalization in the last year, living in the south region (Londrina, state of Paraná, Brazil) and it was associated with ST354 strain after sequencing type analysis.

For genome sequencing, total DNA was extracted using a PureLink^TM^ Quick Gel Extraction Kit (Life Technologies, CA). Libraries were prepared with a NexteraXT library prep kit (Illumina Inc., San Diego, CA). The samples were sequenced via Illumina NextSeq 550 platform (Illumina Inc., San Diego, CA), using 2 × 150-bppaired-end reads. Reads were *de novo* assembled using Unicyler v0.4.0 software (7). Read with a PHRED quality score below 20 were discarded, and adapters were trimmed using TrimGalore v0.6.5 (https://github.com/FelixKrueger/TrimGalore). The *E. coli* ST354 genome was annotated using the Prokaryotic Genome Annotation Pipeline v.3.2 (PGAP/NCBI). Multilocus sequence type (MLST), antimicrobial resistance (AMR) genes, virulence factors and plasmid replicons were predicted using the MLST v2.0, pMLSTv2.0, ResFinderv4.1, FimTyperv1.0, VirulenceFinderv2.0 and PlasmidFinder v2.1 (https://cge.cbs.dtu.dk/services/).^7^^,^^8^

The *E. coli* ST354 strain exhibited as MDR profile by antimicrobial susceptibility testing (Table 1) according to the Clinical and Laboratory Standards Institute guidelines (CLSI, 2020)^9^ and by minimum inhibitory concentration (MIC) for colistin using automated Vitek® 2 system.

**Table 1:** Antimicrobial susceptibility profile of *E. coli* ST354, containing CTX-M-24 and KPC-2 enzymes, obtained from community-acquired urinary tract infection in Brazil.

Antimicrobials	Susceptibility profile	
	Sensitive (S)	Resistant (R)
Ampicillin (AMP)	-	R
Amoxicillin/clavulanate (AMC)	-	R
Trimethoprim-sulfamethoxazole (STX)	S	-
Piperacillin-tazobactam (TZP)	-	R
Cefalexin (CFX)	-	R
Cefuroxime (CXM)	-	R
Ceftriaxone (CRO)	-	R
Cefepime (FEP)	-	R
Meropenem (MEM)	-	R
Ertapenem (ERP)	-	R
Amikacin (AK)	S	-
Gentamicin (CN)	S	-
Ciprofloxacin (CIP)	-	R
Norfloxacin (NOR)	-	R
Nitrofurantoin (F)	S	-
Nalidixic acid (NA)	-	R

*E. coli* ST354 is associated with zoonosis and human infections (10). Generally, this strain causes extra-intestinal infections in humans and other animals. Besides, ST354 strain has a resistant profile to fluoroquinolone.^10^ The virulome of this isolate from Brazil showed a vast repertoire of virulence consisting of *eilA* (Salmonella HilA homologue), *ipfA* (long polar fimbriae), *air* (enteroaggregative immunoglobulin repeat protein), *iss* (increased serum survival) and *gad* (glutamate decarboxylase alphagenes). The presence of these virulence factors provides information regarding the high pathogenicity profile of *E. coli* ST354 lineage isolated from community-acquired urinary tract infection.

Concerning the resistome of *E. coli* ST354, it contains resistance factors to beta-lactam antibiotics. These factors are TEM-1B, CTX-M-24 and KPC-2. The *bla*CTX-M-24 gene is present in the IncF (IncF [F-:A1:B32]) plasmid while *bla*KPC-2 was identified within the IncX3/IncU replicons. Additionally, this strain carries the *tet(B)* gene, which confers resistance to tetracycline. Finally, many point mutations in the *parC* (Ser80Ile, Glu84Gly, Ser57Thr), *parE* (Ile355Thr, Leu416Phe) and *gyrA* (Ser83Leu, Asp87Asn) genes have been identified and are associated with resistance to fluoroquinolones (FQ) drugs. GyrA and ParC proteins are FQ targets. Point alterations in their genes can lead to FQ resistance.^11^ The most frequent substitutions are Ser83Leu and Asp87Asn for *gyrA* and Ser80Ile for *parC* gene.^11^^,^^12^ The presence of resistance factors in replicons and chromosomal DNA reinforces the strong, resistant profile of *E. coli* ST354 strain making it become a potent MDR microorganism.

Many incompatibility group (Inc) plasmids are involved with resistance to several drugs in *E. coli* lineages*.* These replicons carry various combinations of resistance genes, and they can be transferred by conjugation.^13^ IncF and IncX are prevalent plasmids type in *E. coli*. IncX4, for example, is frequently identified as a carrier of resistance genes related to FQ and β-lactam antibiotics resistance.^14^^,^^15^ Moreover, IncX4 has a high frequency of self-transfer (10^−1^-10^−4^).^13^ In this study, the IncF [F-:A1:B32] plasmid harbors the *bla*CTX-M-24 gene and IncX3/IncU carry the *bla*KPC-2 gene.

These findings suggest that the presence of *bla*CTX-M-24, *bla*TEM-1B and *bla*KPC-2 genes in *E. coli* ST354 could be related to the multidrug resistance profile obtained in the antimicrobial susceptibility test. This strain was mainly resistant to beta-lactam antibiotics such as cephalosporins. Moreover, point mutations in *parC, parE* and *gyrA* genes observed in *E. coli* ST354 could influence the antimicrobial resistance profile to FQ antibiotics observed in this study. In addition to this resistant behavior, this strain contains several virulent factors such as *air, gad, eilA, iphA* and *iss*, which shows its high pathogenic genome content.

Our data could help understand the genetic basis of high pathogenicity of the MDR *E. coli* ST354 isolated from community-acquired urinary tract infection in Brazil. The presence of *bla*CTX-M-24, *bla*TEM-1B and *bla*KPC-2 genes, as well as incompatibility plasmids such as IncF [F-:A1:B32] and point mutations in the *parC, parE* and *gyrA* chromosomal genes, may help increase the spectrum of antimicrobial resistance in this microorganism and contribute to its pathogenicity.

## Ethical approval

The study was approved by the Ethics and Research Committee of the State University of Londrina CAAE 56869816.0.000.5231.

## Data availability

Draft whole-genome assembly was deposited in DDBJ/ENA/ GenBank under the SRA accession numberSRR10310377.

## Conflicts of interest

The authors declare no conflicts of interest.
